# Predominant Bacteria Detected from the Middle Ear Fluid of Children Experiencing Otitis Media: A Systematic Review

**DOI:** 10.1371/journal.pone.0150949

**Published:** 2016-03-08

**Authors:** Chinh C. Ngo, Helen M. Massa, Ruth B. Thornton, Allan W. Cripps

**Affiliations:** 1 School of Medical Science, Griffith University, Gold Coast, Queensland, Australia; 2 Molecular Basis of Disease, Menzies Health Institute Queensland, Griffith University, Gold Coast, Queensland, Australia; 3 School of Paediatrics and Child Health, University of Western Australia, Perth, Western Australia, Australia; 4 Telethon Kids Institute, University of Western Australia, Perth, Western Australia, Australia; Ross University School of Medicine, DOMINICA

## Abstract

**Background:**

Otitis media (OM) is amongst the most common childhood diseases and is associated with multiple microbial pathogens within the middle ear. Global and temporal monitoring of predominant bacterial pathogens is important to inform new treatment strategies, vaccine development and to monitor the impact of vaccine implementation to improve progress toward global OM prevention.

**Methods:**

A systematic review of published reports of microbiology of acute otitis media (AOM) and otitis media with effusion (OME) from January, 1970 to August 2014, was performed using PubMed databases.

**Results:**

This review confirmed that *Streptococcus pneumoniae* and *Haemophilus influenzae*, remain the predominant bacterial pathogens, with *S*. *pneumoniae* the predominant bacterium in the majority reports from AOM patients. In contrast, *H*. *influenzae* was the predominant bacterium for patients experiencing chronic OME, recurrent AOM and AOM with treatment failure. This result was consistent, even where improved detection sensitivity from the use of polymerase chain reaction (PCR) rather than bacterial culture was conducted. On average, PCR analyses increased the frequency of detection of *S*. *pneumoniae* and *H*. *influenzae* 3.2 fold compared to culture, whilst *Moraxella catarrhalis* was 4.5 times more frequently identified by PCR. Molecular methods can also improve monitoring of regional changes in the serotypes and identification frequency of *S*. *pneumoniae* and *H*. *influenzae* over time or after vaccine implementation, such as after introduction of the 7-valent pneumococcal conjugate vaccine.

**Conclusions:**

Globally, *S*. *pneumoniae and H*. *influenzae* remain the predominant otopathogens associated with OM as identified through bacterial culture; however, molecular methods continue to improve the frequency and accuracy of detection of individual serotypes. Ongoing monitoring with appropriate detection methods for OM pathogens can support development of improved vaccines to provide protection from the complex combination of otopathogens within the middle ear, ultimately aiming to reduce the risk of chronic and recurrent OM in vulnerable populations.

## Introduction

Otitis media, may be simply defined as inflammation of the middle ear, and is the most common reason a child under the age of 5 will visit their general practitioner and be prescribed antibiotics in socioeconomically developed countries [1]. OM has a range of clinical presentations including AOM, which is characterised by the rapid onset of local and systemic symptoms, including otalgia, fever, vomiting and accumulation of fluid in the middle ear cavity and OME, where the child experiences MEF accumulation without the systemic symptoms. Both of these presentations may occur recurrently or chronically [2]. Globally, more than 700 million cases of AOM are diagnosed each year, with 50% of affected children being under five years of age [3]. Recurrent AOM (RAOM) occurs where a patient has 3 diagnosed AOM episodes within six months or more than 4 episodes in 12 months [4] and is commonly observed in up to 65% of children by 5 years of age[4] OME typically resolves spontaneously within 3 months [5] however, 30–40% of children experience persistent or chronic fluid in the middle ear for more than 3 months (chronic OME, COME) and may require surgical intervention to aid resolution [5].

Irrespective of clinical presentation, OM is a multi-factorial disease, with many associated risk factors, including environmental, immunological deficiency, gender, age and microbial exposure [4, 6]. Despite this complexity, bacterial and viral pathogens, individually and together, are strongly associated with OM development, for example, only 4% children diagnosed with AOM had no bacterial or viral pathogen detected using culture and PCR [7].

The three bacteria most frequently identified in association with OM are: *S*. *pneumoniae*, *H*. *influenzae* and *M*. *catarrhalis* [6], whilst the viruses most commonly associated with OM are respiratory syncytial virus, adenovirus, rhinovirus and coronavirus [6].

Monitoring both the identification and frequency of detection of common otopathogens in OM is central to evaluation of the effects of treatment and impact of vaccination programs. For example, pneumococcal carriage was reduced in association with the introduction of the heptavalent pneumococcal conjugate vaccine (PCV7) in the US and has also increased prevention of early AOM infections [8].

Implementation of PCV7 into national immunisation programs (NIPs) has also resulted in shifts to non-vaccine pneumococcal serotypes isolated from invasive pneumococcal disease and OM [9–12]. Importantly, PCV7 implementation has resulted in *non-typeable (non-encapsulated) H*. *influenzae* (NTHi) detection frequency surpassing *S*. *pneumoniae* detection in AOM patients within a number of countries including the US [12], Spain [13] and France [14]. The emergence of non-vaccine serotypes and their potential role as pathogens in OM is an area of ongoing research interest.

This systematic review examines the frequency of detection of bacteria within MEF from children experiencing OM, globally. Ongoing surveillance of bacteria predominant in OM patients, improved detection methods such as PCR and monitoring the impact of immunisations will continue to inform treatment decisions and ultimately vaccine development aimed to prevent OM development in young children.

## Methods

### Data sources and searches

The design and construction of this systematic review was informed and in compliance with PRISMA (2009) [15] (see S1 PRISMA Checklist). Published literature, including conference abstracts, was searched via PubMed database and only original articles with an English abstract were retrieved and reviewed. Publication dates were restricted from January 1^st^, 1970 to August 30^th^, 2014, with five different search strategies used, as detailed below.

### PubMed search strategy

The following terms were used in five literature searches:

- Search 1—Asian region: (otitis media (Asia (aetiology, otopathogens, pathogens, microbiology, bacteriology, bacteria))) or (otitis media (Bangladesh, Brunei, Cambodia, China, India, Indonesia, Iran, Israel, Japan, Korea, Laos, Lebanon, Malaysia, Pakistan, Philippines, Saudi Arabia, Singapore, Taiwan, Thailand, Turkey, Vietnam)) (S1 Fig)
- Search 2—American region: (otitis media (America (aetiology, otopathogens, pathogens, microbiology, bacteriology, bacteria))) or (otitis media (Argentina, Brazil, Chile, Colombia, Costa Rica, Cuba, Dominica Republic, Ecuador, Honduras, Mexico, Panama, Paraguay, Uruguay, Venezuela, Canada, The US)) (S2 Fig)
- Search 3—African region: (otitis media (Africa (aetiology, otopathogens, pathogens, microbiology, bacteriology, bacteria))) or (otitis media (Algeria, Cameroon, Cote D’lvoire, Egypt, Mozambique, Namibia, Nigeria, South Africa, Zambia, Zimbabwe)) (S3 Fig)
- Search 4—European region: (otitis media (Europe (aetiology, otopathogens, pathogens, microbiology, bacteriology, bacteria))) or (otitis media (Britain, Denmark, England, France, Finland, Germany, Greece, Ireland, Italy, Netherlands, Norway, Poland, Portugal, Romania, Russia, Scotland, Spain, Sweden, Ukraine, Wales, The UK)) (S4 Fig)
- Search 5—Oceanian region: (otitis media (Oceania (aetiology, otopathogens, pathogens, microbiology, bacteriology, bacteria))) or (otitis media (Australia, Cook Islands, New Zealand, Papua New Guinea, Solomon Islands)) (S5 Fig)

### Selection criteria

All articles in English were assessed for relevance by review of abstracts when available, prior to full review of each article. Pre-determined inclusion selection criteria were used: (i) only human studies (ii) age range one month– 18 years (majority of reported study populations below 8 years old) (iii) otopathogenic bacteria were identified from MEF samples of children experiencing a range of presentations of OM, including AOM/RAOM/AOMTF or OME/COME but not chronic suppurative OM (CSOM) (iv) MEF samples were collected through clinical perforation of the tympanic membrane (myringotomy or tympanocentesis) only, rather than spontaneous perforation.

### Study selection and data extraction

All articles meeting the keyword criteria, identified in the primary database search, were screened independently for relevancy by two of the authors (C.C.N. and H.M.M) who read all article titles and abstracts in a standardised manner. Abstracts and articles were reviewed and compared to the inclusion criteria above and key data evaluating the reported details of the study population (age), types of OM, sample size, sample collection methods, microbiological method and years of study were recorded for included studies. Furthermore, where the detail of the sample collection method or clinical criteria for diagnosis of type of OM was inconclusive, the full paper was retrieved and reviewed to determine inclusion. Discussion between reviewers resolved any disagreement regarding inclusion. Additional information was sought from the authors of included reviews, via emailed request seeking additional unpublished information regarding vaccination status and/or serotyping data, if available for the study populations, or to clarify information, such as sampling methods used in their published study. All bibliographies of the included studies were manually searched for additional relevant references.

### Data items, synthesis and analysis

Data illustrating the frequency of detection of predominant bacteria from each report were collated and recorded. The antibiotic sensitivity of these bacteria and the vaccination status of the participants were recorded, where available. Data tables within this narrative report were compiled from the primary results from each included report. Meta-analysis was not performed due to variation of the designs of the included studies and study population characteristics within the published reports.

### Evaluation of study quality

In this study, included studies were previously published in peer reviewed journals. The variety of individual study designs prevented use of a validated instrument to assess the quality of included studies. The assessment of risk of bias of individual studies was performed at the study level by the use of inclusion criteria. For example, clinical criteria describing the varied presentations of OM may have varied over the thirty years of published literature reviewed. Reviewing authors ensured that all published studies clearly differentiated acute AOM by the presence of otalgia and inflamed and/or bulging tympanic membrane which remain consistent characteristics within current definitions. Where vaccination status of the study population is unknown, comparison of the study dates versus the implementation of the NIPs was used to inform potential vaccination status. Only studies that sampled the MEF after clinical perforation of the tympanic membrane were included in this report. This criterion is likely to have generated selection bias on the basis of MEF collection being performed primarily for research purposes, rather than clinical or treatment reasons. The likely outcome would result in skewing representation to include more severe cases undergoing surgical treatment. In addition, there is a relative paucity of reports from some world regions.

## Results

**Fig 1:**
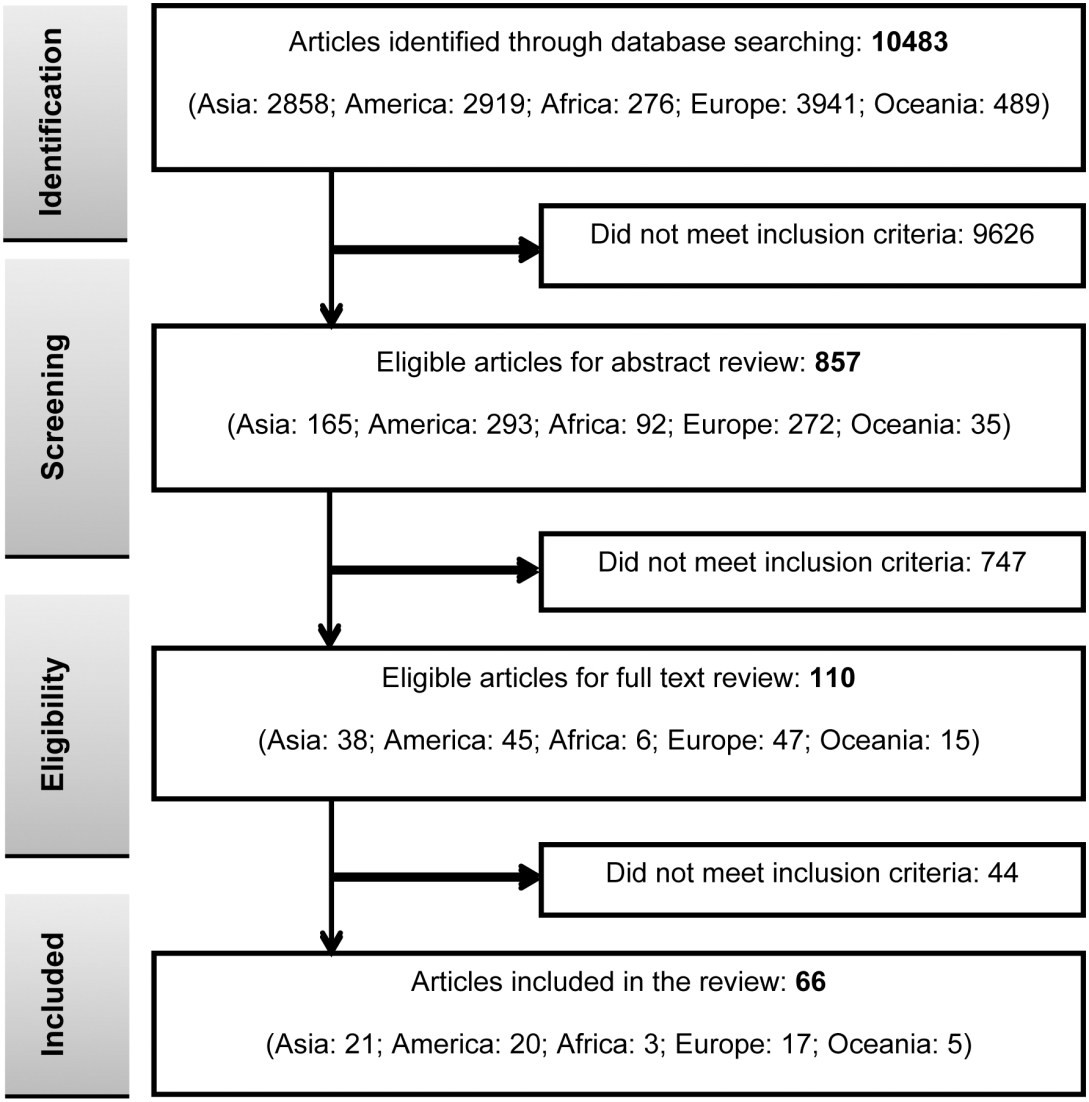
Literature search and selection.

The search “otitis media and children” identified 9,617 articles via PubMed database search over the period January 1^st^, 1970 to August 30^th^, 2014. After performing the key word screening for each of the 5 regions, as illustrated in Fig 1, 10,483 articles were identified, screened for eligibility and 857 abstracts and 110 full publications were reviewed. Sixty-six articles met all the previously described criteria. The number of articles varied for each of the 5 regions, studies reporting from the Americas (n = 20), Europe (n = 17), Asia (n = 21), Africa (n = 3) and Oceania (n = 5) (Fig 1). Within the Americas, European and Asian regions, the reports analysed described 8 different countries within each region. In contrast, within the African and Oceania regions, reports were restricted to only two countries each; South Africa and Egypt and Australia and New Zealand, respectively.

Clinical variants of OM presentation included in this review are AOM (n = 38 studies; 58%), OME/COME (n = 24; 36%), RAOM/AOMTF (n = 9; 14%) and RAOM/OME (n = 1, 2%) within the included studies (n = 66). This percentage is greater than 100% due to 4 studies, in which, more than one type of OM was investigated [16–19]. All studies included in this review, reported microbiological findings from MEF collected via clinical perforation of the tympanic membrane.

### Microbiology of AOM, RAOM/AOMTF and COME

#### Bacterial isolation overview

This systematic review includes 66 reports of bacterial isolation, using culture techniques, from MEF samples of patients experiencing AOM (n = 38, mean percentage of samples positive for bacteria 62%; range 25%–95%), RAOM/AOMTF (n = 12; mean percentage of samples positive for bacteria 45%, range 27%- 68%) and OME/COME (n = 24, mean percentage of samples positive for bacteria 36%; range 14%-73%) from across the world. Eight studies reported bacterial identification for more than one clinical presentation of OM. Bacterial isolation and identification were performed using PCR and bacterial culture in 17 reports, with only 2 reports from AOM patients [18, 20] rather than RAOM, OME or COME patients.

The wide variety of bacteria isolated in each of the reviewed studies, from patients with AOM, RAOM/AOMTF and OME/COME are shown in S1–S3 Tables, respectively. The frequency of detection of *S*. *pneumoniae*, *H*. *influenzae* and *M*. *catarrhalis* in these reports, identify them as the three predominant bacterial pathogens. Other bacteria isolated from the MEF included: *Streptococcus pyogenes* or Group A Streptococcus, *Staphylococcus aureus*, *Pseudomonas aeruginosa*, *Staphylococcus epidermidis*, *Chlamydia trachomatis*, *Alloiococcus otitidis*, *Klebsiella pneumoniae*, *Escherichia coli*. The variety of microbiota isolated and identified in these reports clearly varied with the experimental design, aims and methodology of each study and thus precludes direct comparisons of the frequency of individual pathogen detection. Thus, this review is focussed on the relative frequency of the predominant bacterial pathogens.

Previous reports for the different clinical presentations were collated and examined regionally. Identification of the three predominant bacteria within the MEF for children experiencing AOM, RAOM/AOMTF and OME/COME are presented in S1–S3 Tables, respectively. These tables summarise the published reports of bacteria isolated and identified from MEF using bacterial culture (S1–S3 Tables).

#### AOM

The bacterium most frequently identified from the MEF from children diagnosed with AOM, from previous reports across the world, was *S*. *pneumoniae* closely followed by *H*. *influenzae* (see Table 1). On average, *S*. *pneumoniae* detection was significantly higher than *H*. *influenzae* when all previous studies were pooled (P<0.036 2-way ANOVA without replication) and these reports included samples collected between 1979 and 2010.

**Table 1:** *S*. *pneumoniae*, *H*. *influenzae* and *M*. *catarrhalis* detected from MEF of patients with AOM.

Countries/regions	OM	Age^a^	Size	Bacteria^b^			Ref.
				Hi	Spn	Mcat	
**South America**							
Argentina (1990–1992)	AOM	1m—11y	161	13.7%	20.5%	1.2%	[27]
Argentina (1996–1997)	AOM	15d—24m	367	24.5%	27.8%	0.5%	[28]
Brazil (1990–1995)	AOM	2m—5y	300	7.0%	16.0%	5.0%	[29]
Chile (1998–1999)	AOM	3m—9y	170	24.1%	37.1%	1.2%	[30]
Chile (1998–2002)	AOM	3m—9y	543	28.7%	39.8%	4.2%	[31]
Costa Rica (1992–1997)	AOM	4m—12y	398	14.1%	29.6%	1.5%	[32]
Costa Rica (1999–2001)	AOM	4m—12y	102	13.7%	42.2%	2.9%	[16]
Costa Rica (2002–2007)	AOM	2m—8y	880	23.0%	27.7%	8.8%	[17]
Colombia (1979–1985)	AOM	18d—11y	111	35.1%	23.4%	0.9%	[33]
Colombia (2008–2009)	AOM	3m—5y	83	28.9%	30.1%	ND^c^	[34]
Mexico (2008–2009)	AOM	3m—5y	99	31.3%	31.3%	2.0%	[35]
Venezuela (2008–2009)	AOM	3m—5y	82	41.5%	23.2%	1.2%	[36]
Average				**23.8%**	**29.1%**	**2.7%**	
Max				41.5%	42.2%	8.8%	
Min				7.0%	16.0%	0.5%	
**North America**							
The US (1989–1993)	AOM	2m—7y	815	23.3%	24.9%	15.0%	[21]
The US (1993–1995)	AOM	≤ 6y (86%)	159	28.3%	38.4%	25.8%	[22]
The US (1989–1998)	AOM	2m—7y	982	23.3%	25.8%	13.1%	[23]
The US (2005–2009)	AOM	2m—3y	184	31.5%	30.4%	7.6%	[24]
The US (2006–2008)	AOM	6m—3y	170	31.8%	20.6%	7.6%	[25]
The US (2008–2010)	AOM	4m—3y	208	28.4%	31.7%	13.9%	[26]
Average				**27.8%**	**28.6%**	**13.8%**	
Max				31.8%	38.4%	25.8%	
Min				23.3%	20.6%	7.6%	
**Europe**							
Spain (1989–1995)	AOM	1m—14y	104	28.8%	34.6%	1.0%	[37]
Finland (1980–1985)	AOM	≤ 3m	85	11.8%	18.8%	3.5%	[38]
Finland (1990–1992)	AOM	3m—8y	118	18.6%	22.0%	10.2%	[20]
Finland (1994–1995)	AOM	2m—2y	772	22.5%	26.0%	22.9%	[39]
Finland (1998–1999)	AOM	7m—6y	79	29.1%	49.4%	27.8%	[40]
Germany (2008–2010)	AOM	3m—5y	24	29.1%	ND	4.2%	[41]
Average				**23.3%**	**30.2%**	**11.6%**	
Max				29.1%	49.4%	27.8%	
Min				11.8%	18.8%	1.0%	
**Asia**							
Israel (1995–1996)	AOM	3m—3y	249	45.4%	37.8%	4.8%	[42]
Israel (1995–1999)	AOM	<2m	137	29.9%	40.9%	2.2%	[43]
Israel (1996–2003)	AOM	3m—3y	771	54.6%	35.9%	1.2%	[44]
Japan (1979–1980)	AOM	≤ 16y	406	32.0%	49.9%	ND	[45]
Japan (2001–2002)	AOM	1m—9y	81	7.4%	9.9%	7.4%	[46]
Japan (2003)	AOM	Children	138	21.7%	21.0%	3.6%	[47]
Japan (2002–2004)	AOM	≤ 10y	1092	20.8%	22.3%	4.4%	[48]
Japan (2006)	AOM	9m—8y	40	5.0%	12.5%	ND	[18]
Taiwan (2004)	AOM	4m—13y	96	13.5%	16.7%	ND	[49]
Thailand (2008–2009)	AOM	3m—5y	107	17.8%	24.3%	6.5%	[50]
Turkey (1998–2000)	AOM	6m—10y	78	14.1%	23.1%	5.1%	[51]
Turkey (2002–2004)	AOM	6m—12y	180	13.3%	25.6%	10.0%	[52]
Turkey (2003–2004)	AOM	6m—12y	120	13.3%	23.3%	8.3%	[53]
Average				**22.2%**	**26.4%**	**5.4%**	
Max				54.6%	49.9%	10.0%	
Min				5.0%	9.9%	1.2%	
**Africa**							
South Africa (1999)	AOM	2m—7y	173	5.2%	20.20%	1.20%	[54]
Average (all regions)				**23.1%**	**27.8%**	**7.0%**	
Max (all regions)				54.6%	49.9%	27.8%	
Min (all regions)				5.0%	9.9%	0.5%	

^a^: d = day; m = month; y = year;

^b^: Hi: *H*. *influenzae*; Spn: *S*. *pneumoniae*; Mcat: *M*. *catarrhalis;*

^c^ ND: Not detected

Regionally, in MEF from AOM patients, the average frequency of *S*. *pneumoniae* was higher than NTHi detection for all regions, although there was no significant difference between average detection frequencies for these bacteria in the US (27.8% NTHi versus 28.6% *S*. *pneumoniae*). In contrast, NTHi was the predominant bacteria, comparing average frequencies of bacterial detection for all 3 regions that reported RAOM/AOMTF; South America, Europe and Oceania. Two studies conducted in Costa Rica showed that *S*. *pneumoniae* was predominant in RAOM.

Within each world region, the trend toward more frequent detection of *S*. *pneumoniae*, that is, the average frequency of *S*. *pneumoniae* was higher than NTHi detection for all regions. Indeed, where multiple reports were available for the same country within a region, reports from a number of countries including Finland, Colombia, USA and Japan showed changes in the predominant bacteria identified. Multiple studies from the USA provide evidence of temporal trends in the detection frequency changing from *S*. *pneumoniae* in studies recruiting between 1989–1998 [21–23] to *H*. *influenzae* for similar studies recruiting between 2005–2009 [24, 25], then back to *S*. *pneumoniae* 2006–2010 [26] (Table 1).

For children experiencing AOM (Table 1), 29/38 of studies reported *S*. *pneumoniae* as the predominant bacteria compared to *H*. *influenzae* (primarily non-typeable) being clearly predominant bacteria in 6/38 studies (equivalent detection in 3 reports). The overall average frequency of detection for all studies was 27.8% (range 9.9%-49.9%) for *S*. *pneumoniae* compared to 23.1% (range 5.0%-54.6%) for *H*. *influenzae*, *M*. *catarrhalis* was detected in 34/38 studies at an average frequency of detection of 7.0% (range 0.5%–27.8%). Although detected less frequently overall, *M*. *catarrhalis* frequency, does not demonstrate a consistent temporal trend toward increasing detection in reports of AOM. Individual countries such as Finland, Costa Rica and Chile exhibit a temporal trend toward increasing rates of identification. In contrast, reports from the USA, Japan and Turkey show no consistent trend in identifications whilst reports from Israel do not support any temporal change in *M*. *catarrhalis* detection over time. Previous reports investigated chronic or longer term OM presentations such as RAOM/AOMTF (Table 2) or OME/COME (Table 3).

**Table 2:** *S*. *pneumoniae*, *H*. *influenzae* and *M*. *catarrhalis* detected from MEF of patients with RAOM/AOMTF.

Countries	OM	Age^a^	Size	Bacteria^b^			Ref.
				Hi	Spn	Mcat	
**South America**							
Costa Rica (1999–2001)	RAOM	4m—11y	98	12.2%	32.7%	ND^c^	[16]
Costa Rica (1999–2001)	AOMTF	4m—11y	76	19.7%	17.1%	ND	[16]
Costa Rica (2002–2007)	RAOM	2m—8y	138	26.1%	29.0%	3.6%	[17]
Costa Rica (2002–2007)	AOMTF	2m—8y	90	24.4%	21.1%	4.4%	[17]
**Europe**							
France (2007–2009)	RAOM/ AOMTF	3m—3y	143^d^	31.5%	31.5%	1.4%	[55]
Netherlands (2008–2009)	RAOM	7m—6y	25	24.0%	ND	4.0%	[19]
Spain (2008–2010)	RAOM/ AOMTF	3m—3y	77	41.6%	16.9%	1.3%	[13]
**Oceania**							
Australia (2007–2009)	RAOM	7m—3y	143	13.3%	5.6%	6.3%	[56]
Australia (2007–2009)	RAOM	9m—3y	38	15.8%	7.9%	ND	[57]
New Zealand (2011)	RAOM/OME	3m—3y	325^e^	19.4%	8.0%	8.0%	[58]
Average (all regions)				22.8%	18.9%	4.1%	
Max (all regions)				41.6%	32.7%	8.0%	
Min (all regions)				12.2%	5.6%	1.3%	

^a^ d = day; m = month; y = year;

^b^ Hi: *H*. *influenzae*; Spn: *S*. *pneumoniae*; Mcat: *M*. *catarrhalis;*

^c^ ND: Not detected;

^d^ 18% from spontaneous otorrhoea;

^e^ More than 60% of patients had RAOM

**Table 3:** *S*. *pneumoniae*, *H*. *influenzae* and *M*. *catarrhalis* detected from MEF of patients with OME/COME.

Countries/regions	OM	Age	Size	Bacteria*			Ref.
				Hi	Spn	Mcat	
**Americas**							
Brazil (2001–2002)	OME/COME	11m—10y	128	10.2%	6.2%	3.9%	[59]
The US (1995)	COME	9m—15y	97	21.6%	5.2%	5.2%	[60]
Average				**15.9%**	**5.7%**	**4.6%**	
Max				21.6%	6.2%	5.2%	
Min				10.2%	5.2%	3.9%	
**Europe**							
Finland (1981)	COME	5m—15y	110	3.6%	2.7%	0.9%	[61]
Wales (1986–1987)	COME	≤ 7y (67%)	259	12.4%	2.7%	0.4%	[62]
England (1989)	COME	< 10y (Mostly)	102	16.7%	3.9%	1.0%	[63]
Finland (1993–1994)	OME	5m—12y	165	8.5%	10.3%	6.7%	[64]
Finland (1993–1994)	OME/COME	< 12y	123	14.6%	11.4%	6.5%	[65]
Finland (1996–1997)	OME	1y—9y	67	9.0%	3.0%	9.0%	[66]
Spain (2007)	OME/COME	1y—12y	40	12.5%	2.5%	ND**	[67]
Netherlands (2008–2009)	COME	< 6y	94	19.1%	5.3%	9.6%	[19]
UK (2012)	COME	≤ 18y (83%)	62	3.2%	6.50%	4.8%	[68]
Average				**11.1%**	**5.4%**	**4.9%**	
Max				19.1%	11.4%	9.6%	
Min				3.2%	2.5%	0.4%	
**Asia**							
Turkey (1999)	COME	2y—14y	37	10.8%	16.2%	2.7%	[69]
Korea (2004–2008)	COME	2y—7y	289	ND	1.7%	ND	[70]
Korea (2000–2002)	COME	1y—11y	278	7.9%	1.4%	ND	[71]
Japan (2006)	COME	9m—8y	76	5.3%	1.3%	1.3%	[18]
Japan (1988)	OME	≤ 15y (73%)	613	5.7%	3.3%	0.5%	[72]
Iran (2007–2008)	COME	2y—13y	63	9.5%	15.9%	9.5%	[73]
Iran (2009–2010)	COME	1y—12y	63	4.8%	9.5%	9.5%	[74]
Lebanon (1996–1997)	OME	2y—10y	47	19.1%	ND	4.3%	[75]
Lebanon (2009–2010)	COME	< 13y	107	19.6%	8.4%	3.7%	[76]
Average				**10.3%**	**7.2%**	**4.5%**	
Max				19.6%	16.2%	9.5%	
Min				4.8%	1.3%	0.5%	
**Africa**							
Egypt (1993)	COME	15m—8y	104	18.3%	5.8%	8.7%	[77]
Egypt (2003–2008)	OME/COME	3y—10y	50	ND	12.0%	14.0%	[78]
Average				**18.3%**	**8.9%**	**11.4%**	
Max				18.3%	12.0%	14.0%	
Min				18.3%	5.8%	8.7%	
**Oceania**							
Australia (1995–2000)	COME	11m—10y	45	6.7%	ND	4.4%	[79]
New Zealand (1994)	COME	11m—8y	105	16.2%	8.6%	12.4%	[80]
Average				**11.5%**	**8.6%**	**8.4%**	
Max				16.2%	8.6%	12.4%	
Min				6.7%	8.6%	4.4%	
Average (all regions)				**11.60%**	**6.5%**	**5.7%**	
Max (all regions)				21.6%	16.2%	14.0%	
Min (all regions)				3.2%	1.3%	0.4%	

^a^ d = day; m = month; y = year;

^b^ Hi: *H*. *influenzae*; Spn: *S*. *pneumoniae*; Mcat: *M*. *catarrhalis;*

^c^ ND: Not detected

Examination of the RAOM/AOMTF reports (n = 7 and n = 3, respectively) did not demonstrate a predominant bacteria overall (Table 2) although the average bacterial detection frequency for *H*. *influenzae* was demonstrated as 22.8% (range 12.2%-41.6%) in comparison to 18.6%; (range 5.6%-32.7%) for *S*. *pneumoniae*. *M*. *catarrhalis* detection was very low (not detected in 3 of 10 reports) and average detection frequency from bacterial culture was 4.1% (range 1.3%-8%). There were no significant regional or temporal trends observed, due to the paucity of studies and the challenge arising from recruitment of overlapping clinical presentations (RAOM/AOMTF) within different study designs.

#### OME/COME

Overall, MEF samples from patients diagnosed with OME/COME were less likely to be culture positive for the 3 predominant bacteria in comparison to MEF samples from patients diagnosed with AOM. This is evidenced by the average frequency of detection for *H*. *influenzae* and *S*. *pneumoniae* from OME/COME patients being approximately half that reported from AOM patients (11.6% vs 23% and 6.5% vs 27%) for these bacteria respectively.

Globally, *H*. *influenzae* was the predominant bacteria identified within the MEF of patients experiencing OME/COME (P<0.001 2-way ANOVA without replication) with the average detection frequency was 11.6% (range 3.2%-21.6%) compared to *S*. *pneumoniae* detection 6.5% (range 1.3%-16.2%).

Regionally, *H*. *influenzae* was identified most frequently from patients with OME/COME in South and North America, Europe, Asia, Africa and Oceania however in the Asian and European regions, four of the nine (44%) and two of nine (22%) of studies reported that *S*. *pneumoniae* was predominant, respectively.

Within this review, the bacterial pathogens associated with OM in the MEF were examined regionally and evidence of development of antibiotic resistance recorded. Where resistance was reported, it was included in the summary for each region provided below.

### Regional variation of *H*. *influenzae* strains and Antibiotic sensitivity

Overall, there is a general paucity of reports in which *H*. *influenzae* strains and antibiotic sensitivity have been reported from the MEF of OM patients. In general, the available reports highlight the high proportion of NTHi isolates compared to other *H*. *influenzae* strains identified from AOM patients across almost all world regions; the Americas, Europe, Asia and Oceania, with no evidence available from Africa. The proportion of *H*. *influenzae* isolates from AOM patients that were β-lactamase positive ranged less than 20% for most regions to over 83% in a report from Mexico [35] with no consistent patterns recognisable in regions where multiple reports were available. *H*. *influenzae* isolates from COME patients generally reported a proportion of β-lactamase positive strains. The majority of reports from across the world identified very high proportions of *M*. *catarrhalis* strains as β-lactamase positive.

In South America, AOM-associated *H*. *influenzae* strains isolated within South America are predominantly NTHi, ranging from 63% [36] to 100% [17, 35] and *H*. *influenzae* types a, b, c, d and f which were identified in AOM patients in Chile [30] and Venezuela [36]. Less than 20% of all AOM-associated *H*. *influenzae* strains were β-lactamase positive [17, 28–30, 34, 36]. In contrast, a recent study from Mexico reported that 83% of AOM-associated *H*. *influenzae* strains were β-lactamase positive [35], whilst other reports demonstrated that 100% of AOM-associated *M*. *catarrhalis* strains were β-lactamase positive [17, 29].

In North America, all AOM-related *H*. *influenzae* strains reported were non-typeable [81], with 20%-50% of these strains being β-lactamase positive [24, 81]. Furthermore, 100% of *M*. *catarrhalis* strains isolated from AOM patients were β-lactamase positive [81, 82]. Recently, two studies have reported that *H*. *influenzae* was the bacteria detected most often in MEF from children with AOM since the introduction of pneumococcal vaccine into the NIP [24, 25].

In Europe, a recent study from Germany, reported 86% of AOM-related *H*. *influenzae* strains were non-typeable, with *H*. *influenzae* type b and f also being identified in low frequencies (<10%) [41]. In France, more than 15% of AOM-associated *H*. *influenzae* strains isolated were β-lactamase positive [14, 55] and 95% of AOM-related *M*. *catarrhalis* strains were β-lactamase positive [14].

β-lactamase positive strains of *H*. *influenzae* were reported for 12% and 40% of strains isolated from OME/COME patients in England [63] and Spain [67], respectively.

In Asia, more than 80% of *H*. *influenzae* strains associated with AOM in this region were non-typeable and *H*. *influenzae* type a and b were identified in low frequencies (<20%) [52, 53, 83, 84], except two studies in Thailand [50] and Taiwan [49] where type b was reported in 63% and 100% of *H*. *influenzae* strains. Less than 20% of isolated *H*. *influenzae* strains were β-lactamase positive [43, 52, 53, 83, 85]. In contrast, all OM-associated *M*. *catarrhalis* strains isolated in Israel [43, 83, 85] and over 50% of strains isolated in Turkey [52, 53] were β-lactamase positive. Furthermore, more than 50% of *H*. *influenzae* isolates from children with COME in Lebanon were β-lactamase-negative ampicillin-resistant strains [76], however these strains were not typed.

In Africa, there are no reports of *H*. *influenzae* type from AOM patients in the African region, however a single report from Egypt confirmed that more than 80% and 60% of COME-associated *H*. *influenzae* and *M*. *catarrhalis* strains isolated were β-lactamase positive, respectively [77].

In Oceania, In Oceania, *H*. *influenzae* isolates from RAOM patients in Australia were all non-typeable strains with 17% of these strains were β-lactamase positive [56]. Similarly, in New Zealand, 95% of OM-related *H*. *influenzae* strains were non-typeable [58] and a single report of COME-associated *H*. *influenzae* strains in New Zealand reported that 6% (1/17) were type b and 94% (16/17) were not type b [80].

### Changing microbial pattern pre- and post-PCV7 introduction

PCV7 has been introduced into NIPs since the year 2000. For a number of regions, including South America, Asia and Africa, this vaccine has either not yet been introduced to the NIP or only been recently introduced. For example, PCV7 was introduced into Israel’s NIP and South Africa’s NIP in 2009 [86, 87] or was recommended for high risk groups in Mexico in 2006 and subsequently introduced in to Mexico’s NIP in 2008 [35]. Furthermore, the introduction of PCV7 into NIP or high risk groups within individual countries in a region may also impact the aetiology of OM within that region.

The demonstrated impacts of PCV7 immunisation on the aetiology of OM, since its introduction in 2000 includes: reduction of PCV7 serotype detection in OM, replacement of PCV7 serotypes in AOM by non-vaccine serotypes, particularly serotypes 19A and 3 and changes in dominant otopathogens between *H*. *influenzae* and *S*. *pneumoniae*.

In South America, PCV7 was introduced partially in Costa Rica and Venezuela in 2004 [17, 36, 88] and Mexico in 2006 [35]. The vaccine was implemented in NIP of Costa Rica in 2009 [89] and Mexico in 2008 [35, 89].

The overall rates of pneumococcal OM were reduced in the South American region after PCV7 introduction, as did the ratio of *S*. *pneumoniae* and *H*. *influenzae* isolated from patients with OM. For example, in Costa Rica, the frequency of *S*. *pneumoniae* isolated from patients with OM decreased from 42% (1999–2001) to 28% (2002–2007) after PCV7 introduction in 2004, while the percentage of isolated *H*. *influenzae* increased from 14% (1999–2001) to 23% (2002–2007) [16, 17]. Similarly, in Mexico, the proportion of *S*. *pneumoniae* isolated from patients with OM dropped from 52% (2002–2003) to 31% (2008–2009) [35, 90].

Persistence of PCV7 serotypes was demonstrated in early follow up studies in Costa Rica and Mexico, within 3–4 years of PCV7 introduction, in which PCV7 serotypes were identified in 60% and 40% of pneumococcal isolates from patients with OM respectively in these countries [35, 91].

Emergence of non-vaccine serotypes was observed in both countries, with serotype 3 accounting for 10% of identified pneumococcal isolates in Costa Rica [88, 92] and serotypes 19A, 15B and 28A identified in 23%, 10% and 7% of serotypes from OM patients in Mexico, after PCV introduction [35]. In the longer term, 4 years after PCV7 introduction in Venezuela, the predominant pneumococcal serotype isolated from children with OM was serotype 19A (41% of identified serotypes), followed by other non-PCV7 serotypes such as 6A (9%), 11 (9%) and 15 (9%) [36]. Overall, the emergence of non-PCV7 serotypes was concurrent with an overall reduction in the frequency of pneumococcal isolation from children with OM.

In North America, PCV7 was first introduced in the United States to prevent invasive pneumococcal disease in 2000 and 93.6% of children aged from 19 to 35 months had received at least 3 doses of PCV7 [93].

After PCV7 introduction, the predominant pathogen identified in OM in the US changed from *S*. *pneumoniae* to *H*. *influenzae*. Prior to PCV7 introduction, *S*. *pneumoniae* was the most common bacteria isolated from patients with AOM [22, 23, 82] but 1–3 years following PCV implementation, *H*. *influenzae* emerged as the most common isolates from patients with OM [12, 24].

Changes in the *S*. *pneumoniae* serotypes causing AOM had been observed after PCV7 introduction in the United States. Before PCV introduction, vaccine serotypes accounted for more than 70% of pneumococcal strains isolated from middle ear samples of children with AOM [94]. One to two years post-vaccine introduction, these serotypes accounted for about 50% of OM-associated pneumococcal strains isolated from middle ear samples [94] and six to eight years later they were not observed/reported amongst pneumococcal strains isolated from MEF samples of children with OM [12]. Serotype 19A was the predominant serotype and accounted for 35% of OM-related *S*. *pneumoniae* strains isolated MEF samples [12]. Other non-PCV7 serotypes of *S*. *pneumoniae*, such as 3, 6A, 6C, 15, 23B and 11, were identified with lower frequencies in MEF samples of OM children [12, 94]. In recent years, although the pneumococcal serotypes are non-PCV7, frequencies of *S*. *pneumoniae* and *H*. *influenzae* isolates are equivalent [12].

In Europe, a 2008–2009 survey of 32 countries reported that only 17 countries recommended universal PCV7 immunization in children. The remaining countries had only used the vaccine for groups at high risk of infection or not used the vaccine universally [95]. Impacts of the vaccine on OM aetiology and pneumococcal serotypes identified from OM patients were observed in countries where the vaccine was introduced.

Following PCV7 introduction, the dominant otopathogen changed from *S*. *pneumoniae* to *H*. *influenzae* in France and Greece. In France, prior to the introduction of PCV7, *S*. *pneumoniae* was the otopathogen most commonly identified accounting for 54% of isolates and followed by *H*. *influenzae* (34%), however 1–2 years following PCV7 introduction, *H*. *influenzae* became the dominant bacteria, identified within approximately 46% of isolates and followed by *S*. *pneumoniae* (45%) [14]. This change in pattern was evident until 3–4 years post PCV7 implementation, where *S*. *pneumoniae* was again the most commonly isolated specie with mostly non-PCV7 serotypes [14]. Similarly, in Greece, *S*. *pneumoniae* isolation from the middle ear samples of children with AOM decreased from 42% to 31% post PCV introduction, while *H*. *influenzae* increased slightly from 34% to 37% [96].

Introduction of the PCV7 vaccine resulted in changes in the pneumococcal serotypes isolated from patients with OM. In France, pre-PCV7 vaccine introduction (this period, prior to 2004, is identified by the criterion that the rate of complete vaccination in infants under 2 years of age was below 50%), PCV7 serotypes accounted for more than 50% of *S*. *pneumoniae* associated with OM but 1–4 years post-PCV7 introduction, this decreased to less than 30% [14]. Similarly, in Spain, the percentage of PCV7 serotypes dropped from more than 60% to below 10% of pneumococcal strains isolated from patients with OM [97–99]. Following PCV7 introduction, the frequency of the non-PCV7 serotype 19A, increased from 20% to 50% in patients with OM in France [14] and from 8% to 35% in Spain [98, 99].

In Asia, Singapore and Israel introduced PCV7 into their NIPs in 2009 [86, 100–102]. Studies from Israel reported that PCV7 serotypes accounted for approximately 50% of *S*. *pneumoniae* strains associated with OM prior to the vaccine introduction [102, 103]. No studies have been identified in this review, which report rates following PCV7 introduction on microbial aetiology of OM or from other countries in this region.

In Africa, PCV7 was introduced into the NIP of South Africa in 2009 [87] and Gambia and Rwanda in 2010 [104]. A single study from South Africa examined *S*. *pneumoniae* serotypes pre- PCV7 and showed that PCV7 serotypes accounted for more than 90% of OM-associated *S*. *pneumoniae* [54].

In Nigeria, a shift in the predominant bacteria amongst the three main otopathogens detected from MEF samples of patients with AOM was observed, although information on PCV7 introduction has not been reported. In the early 2000s, *S*. *pneumoniae* was reported as the predominant otopathogen, followed by *H*. *influenzae* and *M*. *catarrhalis* [105], however more recently, *H*. *influenzae* was reported as the predominant otopathogen, followed by *M catarrhalis* and *S*. *pneumoniae* [106].

In Oceania, PCV7 was licenced and recommended for Australian Aboriginal children under 2 years of age and other children at high risk of pneumococcal infection in 2001. This recommendation was extended to all children in Australia in 2005 [107]. Pneumococcal serotypes recovered from middle ear samples showed progressive change after PCV7 immunisation. In 2000–2003, pre-PCV7 introduction, vaccine serotypes accounted for approximately 70% of the pneumococci isolated from ear discharges (ear swabs) of Australian children below 15 years of age [108]. In 2007–2009, 2–4 years post-PCV7 inclusion in NIP, non-PCV7 serotypes replaced the vaccine serotypes being isolated from middle ear samples from non-Aboriginal children with OM, accounting for more than 80% of identified serotype, of these serotype 19A made up 40% [56].

In contrast to other regions, *H*. *influenzae* has always been reported as the most common otopathogen isolated from both Aboriginal and non-Aboriginal children with OM in Australia [56, 79, 109–111]. Thus, there has not been a shift between *H*. *influenzae* and *S*. *pneumoniae* as the predominant otopathogen observed pre- and post- PCV7 introduction in the country. Similar observations are reported for New Zealand, with *H*. *influenzae* identified as the most common bacteria isolated from children with OM (RAOM or OME/COME) before and after pneumococcal vaccine introduction (2008)[58, 80, 112].

### Improvement of detection of existing and new otopathogens by PCR

The frequency of bacterial detection within MEF samples from children with OM have been compared using both culture and PCR. Across the world, 17 studies have reported bacterial identification, the majority (n = 15) examined MEF from patients with COME or RAOM, with 2 reports from AOM patients.

Overall, PCR detection methods improved the detection frequency of bacteria although the magnitude of the improvement varied between individual reports and ranged from no difference to 9-fold increase in bacterial detection. On average, the improvement in the frequency of detection for bacteria identified within the MEF of children with OM is 3.2 times higher using PCR compared to bacterial culture, for both *S*. *pneumoniae* and *H*. *influenzae*. The sensitivity of PCR techniques also improved *M*. *catarrhalis* detection by 4.5- fold, on average across all studies (Table 4).

**Table 4:** Comparison of otopathogen detection in MEF using culture and PCR.

Countries	OM	Culture					PCR					Ref.
		Bacteria	Spn	Hi	Mcat	Aot^a^	Bacteria	Spn	Hi	Mcat	Aot	
Brazil (2001–2002)	COME/ RAOM	20%	6%	10%	4%	-	62%	13%	39%	10%	-	[59]
US (1995)	COME	32%	5%	22%	5%	-	131%	30%	55%	46%	-	[60]
Finland (1990–1992)	AOM	51%	22%	19%	10%	ND^b^	58%	20%	11%	27%	25%	[20]
Finland (1993–1994)	COME	33%	11%	15%	7%	ND	131%	35%	33%	63%	20%	[65]
Finland (1996–1997)	OME	21%	3%	9%	9%	ND	76%	21%	18%	37%	46%	[66]
Netherlands (2008–2009)	RAOM	28%	ND	24%	4%	-	84%	13%	42%	29%	-	[19]
Netherlands (2008–2009)	COME	34%	5%	19%	10%	-	63%	7%	32%	24%	-	[19]
Iran (2007–2008)	COME	36%	16%	10%	10%	-	151%	19%	95%	37%	-	[73]
Iran (2009–2010)	COME	25%	10%	5%	10%	24%	32%	11%	11%	10%	40%	[74]
Korea (2000–2002)	COME	9%	1%	8%	ND	-	45%	5%	29%	11%	-	[71]
Turkey (1999)	COME	30%	16%	11%	3%	-	143%	57%	70%	16%	-	[69]
Japan (2005)	COME	10%	ND	5%	5%	-	48%	12%	23%	13%	-	[113]
Japan (2006)	OME	7%	1%	5%	1%	ND	27%	8%	12%	7%	61%	[18]
Japan (2006)	AOM	18%	13%	5%	ND	ND	41%	13%	8%	20%	50%	[18]
Australia (2007–2009)	RAOM	25%	6%	13%	6%	-	66%	6%	47%	13%	-	[56]
Egypt (2003–2008)	OME/ COME	26%	12%	-	14%	-	92%	48%	-	56%	-	[78]
New Zealand (2011)	RAOM/ OME	19%	8%	19%	8%	-	65%	23%	43%	39%	-	[58]
**PCR vs culture** average		**3.3**										
**Hi (PCR vs culture)** average		**3.2**										
**Spn (PCR vs culture)** average		**3.2**										
**Mcat (PCR vs culture)** average		**4.5**										

^a^ Aot: *A*. *otitidis;*

^b^ ND: Not detected

The enhanced sensitivity of detection observed using PCR has resulted in detection of *M*. *catarrhalis* and *S*. *pneumoniae* in culture negative samples [18, 19, 71, 113].

PCR confirmation of the same predominant bacterium being identified within the same sample was observed in 10/17 reports however, in the 6 reports showing a different predominant bacteria, 4/6 changed to *M*. *catarrhalis* and 2/6 changed to *H*. *influenzae*. A predominant bacterium was not identified in one study. Where PCR identified a different predominant bacterium, there was no recognisable pattern compared to the culture result, with 3 changes from *H*. *influenzae* and 3 from *S*. *pneumoniae* as the predominant bacterium from the culture.

Globally, and consistent with the results from bacterial culture, PCR results confirmed overall that *H*. *influenzae* was the most frequently detected bacterium in the MEF of children experiencing COME/RAOM (11/15 studies). *M*. *catarrhalis* was predominant in 3/4 studies and all three bacteria were equally frequent in one study from Iran. The two studies that examined AOM patients MEF, identified *M*. *catarrhalis* most frequently, although *Alloiococcus otitidis* (*A*. *otitidis*) was equally or more frequently identified in these reports.

Regionally, from PCR results, *H*. *influenzae* was detected most frequently in the Americas, Asia and Oceania. In Europe, *H*. *influenzae* and *M*. *catarrhalis* were equally frequent (2/4 studies each).

PCR detection for *A*. *otitidis* from the MEF of OM patients, has been reported from a range of countries, including Turkey [114], Japan [18], Iran [74], Finland [20, 65, 66], Sweden [115], the United Kingdom [116] and the United States [25, 117]. Collectively, *A*. *otitidis* was detected in 20% to 60% of MEF samples from OM patients by PCR, but was largely undetected using culture (Table 4). Furthermore, *A*. *otitidis* was more frequently identified in patients with OME compared to patients with AOM [18, 117] and, was the most commonly detected bacteria in the middle ear of patients with OME/COME, detected using PCR [18, 74, 117, 118]. The use of PCR methodologies has improved detection rates for organisms that are difficult to culture, for example, detection of *A*. *otitidis* within MEF samples from patients with OM requires more than 3 days in culture, thus PCR analysis of MEF samples has improved detection of this organism and has resulted in consideration of *A*. *otitidis* having a role in OM pathogenesis [58, 67, 74, 119].

## Discussion

The frequency of bacterial pathogens of OM, identified within the middle ear fluid using primarily microbial culture, but also PCR, were reviewed over a range of clinical presentations, using reports published since 1970. This review focussed on pathogens actually identified within the MEF rather than extrapolation of upper respiratory tract pathogens coincident in the nasopharynx. Improved understanding of the polymicrobial nature of OM and its varied clinical presentations is informed by the identification of specific bacteria (and viruses) within the middle ear.

Overall, the frequency of bacterial isolation from MEF samples of patients with AOM was generally higher than that from other presentations including RAOM/AOMTF or OME/COME. This finding is consistent with recognition of AOM as an acute, frequently bacterial caused infection, whilst OME and COME are considered as a consequence of a previous AOM episode, or inflammation resulting from the presence of bacterial biofilm or intracellular infection within the middle ear epithelial cell [57]. COME and OME may also result from inflammatory and allergic conditions such as rhino-sinusitis [120].

This review confirms OM as a polymicrobial disease, with the same predominant microbes identified in MEF samples from patients with AOM, RAOM, AOMTF, OME and COME globally. Consistent with previous reports, the three bacteria most frequently identified in the MEF from culture and considered causal for OM were *S*. *pneumoniae*, *H*. *influenzae* and *M*. *catarrhalis*. In Latin American countries, *Streptococcus pyogenes (S*. *pyogenes)* was also frequently identified as a predominant bacterium, pathogenic for OM. Increasingly, A. otitidis has been identified within the MEF using PCR techniques, although this bacterium’s role in OM pathogenesis is still under investigation [121, 122]. Culture based detection of *A*. *otitidis* within MEF samples from patients with OM requires more than 3 days in culture and thus this organism may not have been successfully identified and reported in previous studies [58, 67, 74, 119].

*S*. *pneumoniae* remains the most frequently detected bacteria from patients with AOM in Asia, Africa, America and Europe. No significant differences between the clinical manifestations of AOM, resulting from the different pneumococcal serotypes, were identified but in general, PCV7 serotypes are reported to cause more otalgia or ear ache than non-PCV7-serotypes [123]. Additionally, children with AOM caused by *S*. *pneumoniae* are reported to experience higher fever and more redness of tympanic membrane than those with AOM caused by *H*. *influenzae* or *M*. *catarrhalis* [124], thus increasing the likelihood of presentation for medical treatment. AOM associated with *S*. *pneumoniae* is less likely to resolve without treatment when compared to that caused by *M*. *catarrhalis* [125].

Overall, use of PCV7 has initially reduced the incidence of AOM caused by *S*. *pneumoniae* and altered the serotypes causal for AOM. Replacement of PCV7 serotypes by non-vaccine serotypes has been observed in MEF samples of children with OM in all countries where the vaccine has been used, with serotype 19A, a predominant serotype recovered from vaccinated children with OM. A new genotype of this serotype, isolated from children with AOM was resistant to all USA Food and Drug Administration approved antimicrobial drugs for AOM treatment in children [126]. Therefore, 13-valent pneumococcal conjugate vaccine (PCV13), which includes serotype 19A, may help to reduce the incidence of OM. Since 2010, PCV13 has been licensed and recommended for children in a number of developed countries such as the United States, the United Kingdom, Singapore and Australia [101, 127–129].

In contrast to AOM, *H*. *influenzae* is most frequently detected bacteria in the MEF of patients with RAOM or OME/COME globally and, with the exception of Australia, is the second most common pathogen in patients with AOM across the world. In Australia, *H*. *influenzae* is the predominant bacterium for AOM and OME for indigenous and non-indigenous children [110, 111, 130]. Bilateral AOM, eye symptoms, previous treatment with antibiotics, protracted and recurrent disease, are more likely for AOM *caused by H*. *influenzae* when compared with caused by other pathogens [131, 132].

In most studies included in this review, less than 50% of *H*. *influenzae* strains recovered from patients with OM were β-lactamase producers. β-lactamase is responsible for bacterial resistance to the β-lactam antibiotics, commonly used as the first line of treatment for OM, such as aminopenicillins, cephalosporins, cephamycins and carbapenems [133, 134]. Widespread use of these antibiotics for the treatment of OM has contributed to the development of antimicrobial resistance. β-lactamase-negative ampicillin-resistant (BLNAR) *H*. *influenzae* strains, have been isolated from the MEF of patients with OM in Mexico [35], Lebanon [76] and Japan [135]. The BLNAR strains identified from MEF in the latter two reports [76, 135] have been isolated elsewhere in the upper respiratory tract and reflect the most frequently detected mechanism of ampicillin resistance for *H*. *influenzae* across the world [136]. Although not based on MEF samples, a recent report has described the changing frequency of identification of BLNAR *H*. *influenzae* isolates, both globally and regionally, and confirmed reductions in detection in Europe and Africa between 2004–2008 and 2009–2012 whilst the proportion of detections rose in other regions including Latin America and the Middle East [137].

In this review, NTHi strains were most frequently reported and these strains are unaffected by *H*. *influenzae* b conjugate vaccination alone (Hib) [138]. Increasingly, inclusion of *H*. *influenzae* antigens to improve the opportunity for vaccine development is under development [139].

PCV7 introduction has been associated increased identification of *H*. *influenzae* in patients with OM when compared with *S*. *pneumoniae*. *H*. *influenzae* has gradually replaced *S*. *pneumoniae* as the predominant otopathogen in children with AOM for 1–5 years following PCV7 introduction and, in the absence of an approved vaccine for NTHi prevention, this shift could create another challenge for treatment and prevention of OM.

Until recently, most NTHi vaccine research interest was focussed on the 10-valent pneumococcal NTHi protein D conjugate vaccine (PHiD-CV10), which contains 10 pneumococcal serotypes and protein D of *H*. *influenzae*. This vaccine is immunogenic against protein D and well tolerated in studies conducted in America, Europe, Asia, Africa [140–145] and Australia [129]. Originally considered a potential candidate to replace PCV7, a recent study from Australia demonstrated that 19% of NTHi strains isolated from the nasal cavity, nasopharynx and bronchoalveolar lavage fluid from asymptomatic carriage or children with bronchiectasis were missing the *hpd* genes encoding protein D of NTHi [146]. Further research is needed to trial a number of already identified NTHi vaccine candidates, with respect to the safety, immunogenicity and efficacy against OM [1]. *M*. *catarrhalis* has been recognised as an otopathogen since 1920s with significant increased detection occurring since the 1980’s [147]. Clinically, *M*. *catarrhalis* alone appears to be the least virulent pathogen [148] and is often associated with the first episode of disease, a younger age of onset, lower rates of spontaneous tympanic membrane perforation [149] and increased likelihood of spontaneous resolution [125]. *M*. *catarrhalis* however, most frequently occurs as a polymicrobial infection [149]. In this review, where culture findings of predominant bacteria were identified as different to that identified by PCR methods, *M*. *catarrhalis* was the new predominant bacterium.Most studies report that more than 90% of *M*. *catarrhalis* strains isolated from patients with OM were β-lactamase producers, although lower rates of 40%-50% have been reported from Turkey and Nigeria [52, 53, 106]. Whilst several vaccine candidates have been identified for *M*. *catarrhalis*, development of a vaccine strategy for *M*. *catarrhalis* is at an early stage [150–152].

Otitis media is associated with other bacteria including *S*. *pyogenes* (Group A Streptococcus), the fourth ranked otopathogen [82] identified in this review. This bacterium is otopathogenic and more often identified in older children who experience low rates of fever and respiratory symptoms but, increased rates of spontaneous perforation and mastoiditis, compared with AOM due to other otopathogens [153]. It is speculated that the role of *S*. *pyogenes* in OM aetiology maybe under-recognised in some regions.

Increasingly, new organisms such as *A*. *otitidis*, *Turicella otitidis*, *Pseudomonas otitidis* and *Corynebacterium mucifaciens* have been detected in patients with OM, but their significance in OM pathogenesis remains unclear [154]. Of these organisms, *A*. *otitidis* is the most commonly identified in the MEF from patients with OME/COME [18, 117]. *A*. *otitidis* may be associated with a protracted clinical course, development of the mucoid MEF characteristic of OME [65] and was recently reported to induce inflammation within the middle ear cavity [122, 155] thus potentially contributing as a secondary pathogen in AOM with perforation [156].

In this review, we have reported on the global detection of bacterial otopathogens in the MEF of children experiencing OM. Although three bacteria, *S*. *pneumoniae*, *H*. *influenzae* and *M*. *catarrhalis* dominate, geographical differences are emerging. These differences relate to PCV use, antimicrobial treatment and potentially other local regional factors. This highlights the need for ongoing surveillance and reporting of the microbiology of OM globally, in order to better understand the pathogenesis of this disease and to guide development of appropriate intervention strategies.

## Conclusions

Despite a paucity of comprehensive prospective studies examining the microbial aetiology of AOM/RAOM and OME/COME locally within the middle ear of children throughout the world, we conclude that *S*. *pneumoniae*, *H*. *influenzae* and *M*. *catarrhalis*, have remained remarkably consistent over the past 40 years, as the predominant bacteria causal for OM. Depending on the region, and the detection method, *S*. *pneumoniae* and *H*. *influenzae* vie for predominance as the bacteria most frequently isolated from the middle ear fluid from children with OM. A significant environmental change, the introduction of PCV7 and ongoing monitoring of serotype replacement throughout many regions of the world, continues to provide evidence of the predominance of these two bacteria in children with OM. Importantly, to identify and address the evolving development of bacterial resistance, such as β-lactamase production and potential pathogenicity of prevalent microbes such as *S*. *pyogenes* and *A*. *otitidis* ongoing monitoring is essential.

Future research is essential, whether development of vaccines that include multiple otopathogens [157], bacterial and viral, or trialling the use of probiotic supplementation to the upper respiratory tract [158] for children at high risk of OM. Investigators should continue to aim to minimise the incidence or indeed prevent OM pathogenesis and its long term sequelae for vulnerable children across the world.

## Supporting Information

S1 Fig Strategies for searching studies on pathogens of OM in Asia. (DOCX)

S2 Fig Strategies for searching studies on pathogens of OM in the Americas. (DOCX)

S3 Fig Strategies for searching studies on pathogens of OM in Africa. (DOCX)

S4 Fig Strategies for searching studies on pathogens of OM in Europe. (DOCX)

S5 Fig Strategies for searching studies on pathogens of OM in Oceania. (DOCX)

S1 PRISMA Checklist PRISMA Checklist. (DOCX)

S1 Table Proportion of bacteria detected from MEF samples of patients with AOM. (DOCX)

S2 Table Proportion of bacteria detected from MEF samples of patients with RAOM/AOMTF. (DOCX)

S3 Table Proportion of bacteria detected from MEF samples of patients with OME/COME. (DOCX)
