# Supplementary material for: Predominant Bacteria Detected from the Middle Ear Fluid of Children Experiencing Otitis Media: A Systematic Review
Source: PLoS One. 2016 Mar 8;11(3):e0150949. doi: 10.1371/journal.pone.0150949 (PMC4783106; doi:10.1371/journal.pone.0150949)
Supplement: S1 Fig — (DOCX) [file pone.0150949.s001.docx]

**Figure S1. Strategies for searching studies on pathogens of OM in Asia**

Otitis media

Bangladesh → 13 articles → 0

Brunei → 0

Cambodia → 3 articles → 0

China → 339 articles → 0

India → 276 articles → 0

Indonesia → 5 articles → 0

Iran → 58 articles → 2

Israel → 341 articles → 3

Japan → 774 articles → 7

Korea → 244 articles → 2

Laos → 0

Lebanon → 21 articles → 2

Malaysia → 38 articles → 0

Pakistan → 23 articles → 0

Philippines → 15 articles → 0

Saudi Arabia → 52 articles → 0

Singapore → 36 articles → 0

Taiwan → 165 articles → 1

Thailand → 45 articles → 1

Turkey → 402 articles → 4

Vietnam → 8 articles → 0

Aetiology

n=4

n=0

Otopathogens

n=0

n=0

Microbiology

n=144

n=8

Pathogens

n=30

n=3

Bacteria

n=165

n=8

n=21

(12 AOM; 8 OME/COME; 1 AOM and OME)

Asia
