# Supplementary material for: Predominant Bacteria Detected from the Middle Ear Fluid of Children Experiencing Otitis Media: A Systematic Review
Source: PLoS One. 2016 Mar 8;11(3):e0150949. doi: 10.1371/journal.pone.0150949 (PMC4783106; doi:10.1371/journal.pone.0150949)
Supplement: S2 Fig — (DOCX) [file pone.0150949.s002.docx]

**Figure S2. Strategies for searching studies on pathogens of OM in America**

Otitis media

Argentina → 36 articles → 2

Brazil → 97 articles → 2

Chile → 15 articles → 2

Colombia → 9 articles → 2

Costa Rica → 32 articles → 3

Cuba → 1 article → 0

Dominican Republic → 0

Ecuador → 3 articles → 0

Honduras → 1 article → 0

Mexico → 71 articles → 1

Panama → 5 articles → 0

Paraguay → 1 article → 0

Uruguay → 3 articles → 0

Venezuela → 5 articles → 1

Canada → 328 articles → 0

The US → 2402 articles → 7

Aetiology

n=1

n=0

Otopathogens

n=3

n=1

Microbiology

n=234

n=13

Pathogens

n=72

n=8

Bacteria

n=293

n=13

n=20

(18 AOM/RAOM/AOMTF; 2 OME/COME)

America
