# Supplementary material for: Predominant Bacteria Detected from the Middle Ear Fluid of Children Experiencing Otitis Media: A Systematic Review
Source: PLoS One. 2016 Mar 8;11(3):e0150949. doi: 10.1371/journal.pone.0150949 (PMC4783106; doi:10.1371/journal.pone.0150949)
Supplement: S3 Fig — (DOCX) [file pone.0150949.s003.docx]

**Figure S3. Strategies for searching studies on pathogens of OM in Africa**

Otitis media

Algeria → 1 article → 0

Cameroon → 1 article → 0

Cote D’lvoire → 0

Egypt → 63 articles → 2

Mozambique → 4 articles → 0

Namibia → 1 article → 0

Nigeria → 107 articles → 0

South Africa → 93 articles → 1

Zambia → 3 articles → 0

Zimbabwe → 3 articles → 0

Aetiology

n=6

n=1

Otopathogens

n=0

n=0

Microbiology

n=84

n=3

Pathogens

n=15

n=2

Bacteria

n=92

n=3

n=3

(1 AOM; 2 OME/COME)

Africa
