# Supplementary material for: Predominant Bacteria Detected from the Middle Ear Fluid of Children Experiencing Otitis Media: A Systematic Review
Source: PLoS One. 2016 Mar 8;11(3):e0150949. doi: 10.1371/journal.pone.0150949 (PMC4783106; doi:10.1371/journal.pone.0150949)
Supplement: S4 Fig — (DOCX) [file pone.0150949.s004.docx]

**Figure S4. Strategies for searching studies on pathogens of OM in Europe**

Otitis media

Britain → 105 articles → 0

Denmark → 267 articles → 0

England → 152 articles → 1

France → 346 articles → 1

Finland → 525 articles → 8

Germany → 271 articles → 1

Greece → 110 articles → 0

Ireland → 53 articles → 0

Italy → 347 articles → 0

Netherlands → 397 articles → 2

Norway → 113 articles → 0

Poland → 122 articles → 0

Portugal → 22 articles → 0

Romania → 22 articles → 0

Russia → 32 articles → 0

Scotland → 47 articles → 0

Spain → 182 → 3

Sweden → 380 articles → 0

Ukraine → 4 articles → 0

Wales → 85 articles → 1

The UK → 349 articles → 1

Aetiology

n=14

n=1

Otopathogens

n=1

n=0

Microbiology

n=223

n=5

Pathogens

n=50

n=3

Bacteria

n=272

n=5

n=17

(8 AOM/RAOM; 8 OME/COME; 1 RAOM and COME)

Europe
