# Supplementary material for: Predominant Bacteria Detected from the Middle Ear Fluid of Children Experiencing Otitis Media: A Systematic Review
Source: PLoS One. 2016 Mar 8;11(3):e0150949. doi: 10.1371/journal.pone.0150949 (PMC4783106; doi:10.1371/journal.pone.0150949)
Supplement: S5 Fig — (DOCX) [file pone.0150949.s005.docx]

**Figure S5. Strategies for searching studies on pathogens of OM in Oceania**

Otitis media

Australia → 364 articles → 3

Cook Islands → 5 articles → 0

New Zealand → 111 articles → 2

PNG → 6 articles → 0

Solomon Islands → 3 articles → 0

Aetiology

n=2

n=1

Otopathogens

n=0

n=0

Microbiology

n=35

n=2

Pathogens

n=12

n=2

Bacteria

n=4

n=2

n=4

(2 RAOM; 2 COME; 1RAOM/OME)

Oceania
