# Supplementary material for: Predominant Bacteria Detected from the Middle Ear Fluid of Children Experiencing Otitis Media: A Systematic Review
Source: PLoS One. 2016 Mar 8;11(3):e0150949. doi: 10.1371/journal.pone.0150949 (PMC4783106; doi:10.1371/journal.pone.0150949)
Supplement: S1 Table — (DOCX) [file pone.0150949.s007.docx]

**S1 Table. Proportion of bacteria detected from MEF samples of patients with AOM.**

| **Countries/regions** | **OM** | **Age** | **Size** | **Positive for bacteria** | **Bacteria** | **Ref.** |
| --- | --- | --- | --- | --- | --- | --- |
| ***South America*** |  |  |  |  |  |  |
| Argentina (1990-1992) | AOM | 1m - 11y | 161 | 53% | *S. pneumoniae* | [21] |
|  |  |  |  |  | *H. influenzae* |  |
|  |  |  |  |  | *M. catarrhalis* |  |
|  |  |  |  |  | *S. pyogenes* |  |
|  |  |  |  |  | *S. aureus* |  |
|  |  |  |  |  | *P. aeruginosa* |  |
|  |  |  |  |  | Others |  |
| Brazil (1990-1995) | AOM | 2m - 5y | 300 | 64% | *S. pneumoniae* | [23] |
|  |  |  |  |  | *H. influenzae* |  |
|  |  |  |  |  | *M. catarrhalis* |  |
|  |  |  |  |  | *S. aureus* |  |
|  |  |  |  |  | *P. aeruginosa* |  |
| Argentina (1996-1997) | AOM | 15d - 2y | 367 | 59% | *S. pneumoniae* | [22] |
|  |  |  |  |  | *H. influenzae* |  |
|  |  |  |  |  | *M. catarrhalis* |  |
|  |  |  |  |  | *S. pyogenes* |  |
|  |  |  |  |  | *S. aureus* |  |
|  |  |  |  |  | *P. aeruginosa* |  |
|  |  |  |  |  | Others |  |
| Chile (1998-1999) | AOM | 3m - 9y | 170 | 82% | *S. pneumoniae* | [24] |
|  |  |  |  |  | *H. influenzae* |  |
|  |  |  |  |  | *M. catarrhalis* |  |
|  |  |  |  |  | *S. pyogenes* |  |
|  |  |  |  |  | *S. aureus* |  |
|  |  |  |  |  | *P. aeruginosa* |  |
|  |  |  |  |  | Others |  |
| Chile (1998-2002) | AOM | 3m - 9y | 543 | 87% | *S. pneumoniae* | [25] |
|  |  |  |  |  | *H. influenzae* |  |
|  |  |  |  |  | *M. catarrhalis* |  |
|  |  |  |  |  | *S. pyogenes* |  |
|  |  |  |  |  | Others |  |
| Costa Rica (1992-1997) | AOM | 4mo - 12y | 398 | 59% | *S. pneumoniae* | [26] |
|  |  |  |  |  | *H. influenzae* |  |
|  |  |  |  |  | *M. catarrhalis* |  |
|  |  |  |  |  | *S. pyogenes* |  |
|  |  |  |  |  | *S. aureus* |  |
|  |  |  |  |  | *P. aeruginosa* |  |
|  |  |  |  |  | others |  |
| Costa Rica (1999-2001) | AOM | 4m - 12y | 102 | N/A | *S. pneumoniae* | [16] |
|  |  |  |  |  | *H. influenzae* |  |
|  |  |  |  |  | *M. catarrhalis* |  |
|  |  |  |  |  | *S. pyogenes* |  |
|  |  |  |  |  | *S. aureus* |  |
|  |  |  |  |  | Others |  |
| Costa Rica (2002-2007) | AOM | 2m - 8y | 880 | 57% | *S. pneumoniae* | [17] |
|  |  |  |  |  | *H. influenzae* |  |
|  |  |  |  |  | *M. catarrhalis* |  |
|  |  |  |  |  | *S. pyogenes* |  |
| Colombia (1979-1985) | AOM | 18d - 11y | 111 | 74% | *H. influenzae* | [27] |
|  |  |  |  |  | *S. pneumoniae* |  |
|  |  |  |  |  | *M. catarrhalis* |  |
|  |  |  |  |  | *S. pyogenes* |  |
|  |  |  |  |  | *S. aureus* |  |
|  |  |  |  |  | Others |  |
| Colombia (2008-2009) | AOM | 3m - 5y | 83 | 59% | *S. pneumoniae* | [28] |
|  |  |  |  |  | *H. influenzae* |  |
|  |  |  |  |  | *S. pyogenes* |  |
|  |  |  |  |  | others |  |
| Mexico (2008-2009) | AOM | 3m - 5y | 99 | 64% | *S. pneumoniae* | [29] |
|  |  |  |  |  | *H. influenzae* |  |
|  |  |  |  |  | *M. catarrhalis* |  |
|  |  |  |  |  | *S. pyogenes* |  |
| Venezuela (2008-2009) | AOM | 3m - 5y | 82 | 67% | *H. influenzae* | [30] |
|  |  |  |  |  | *S. pneumoniae* |  |
|  |  |  |  |  | *M. catarrhalis* |  |
|  |  |  |  |  | *S. pyogenes* |  |
| ***North America*** |  |  |  |  |  |  |
| The US (1989-1993) | AOM | 2m -7y | 815 | N/A | *S. pneumoniae* | [31] |
|  |  |  |  |  | *H. influenzae* |  |
|  |  |  |  |  | *M. catarrhalis* |  |
| The US (1993-1995) | AOM | ≤ 6y (86%) | 159 | 95% | *S. pneumoniae* | [32] |
|  |  |  |  |  | *H. influenzae* |  |
|  |  |  |  |  | *M. catarrhalis* |  |
|  |  |  |  |  | *S. pyogenes* |  |
|  |  |  |  |  | *S. aureus* |  |
| The US (1989-1998) | AOM | 2m - 7y | 982 | 71% | *S. pneumoniae* | [33] |
|  |  |  |  |  | *H. influenzae* |  |
|  |  |  |  |  | *M. catarrhalis* |  |
| The US (2005-2009) | AOM | 2m - 3y | 184 | 71% | *H. influenzae* | [34] |
|  |  |  |  |  | *S. pneumoniae* |  |
|  |  |  |  |  | *M. catarrhalis* |  |
|  |  |  |  |  | *S. pyogenes* |  |
|  |  |  |  |  | *P. aeruginosa* |  |
| The US (2006-2008) | AOM | 6m - 3y | 170 | 71% | *H. influenzae* | [35] |
|  |  |  |  |  | *S. pneumoniae* |  |
|  |  |  |  |  | *M. catarrhalis* |  |
|  |  |  |  |  | *S. aureus* |  |
| The US (2008-2010) | AOM | 4m - 3y | 208 | N/A | *S. pneumoniae* | [36] |
|  |  |  |  |  | *H. influenzae* |  |
|  |  |  |  |  | *M. catarrhalis* |  |
| ***Europe*** |  |  |  |  |  |  |
| Spain (1989-1995) | AOM | 1m - 14y | 104 | 74% | *S. pneumoniae* | [37] |
|  |  |  |  |  | *H. influenzae* |  |
|  |  |  |  |  | *M. catarrhalis* |  |
|  |  |  |  |  | *S. aureus* |  |
|  |  |  |  |  | *P. aeruginosa* |  |
|  |  |  |  |  | others |  |
| Finland (1980-1985) | AOM | ≤ 3m | 85 | 84% | *S. pneumoniae* | [38] |
|  |  |  |  |  | *H. influenzae* |  |
|  |  |  |  |  | *M. catarrhalis* |  |
|  |  |  |  |  | *S. aureus* |  |
|  |  |  |  |  | Others |  |
| Finland  (1990-1992) | AOM | 3m - 8y | 118 | 51% | *S. pneumoniae* | [20] |
|  |  |  |  |  | *H. influenzae* |  |
|  |  |  |  |  | *M. catarrhalis* |  |
| Finland (1994-1995) | AOM | 2m - 2y | 772 | 83% | *S. pneumoniae* | [39] |
|  |  |  |  |  | *H. influenzae* |  |
|  |  |  |  |  | *M. catarrhalis* |  |
|  |  |  |  |  | *S. pyogenes* |  |
|  |  |  |  |  | *S. aureus* |  |
|  |  |  |  |  | *P. aeruginosa* |  |
|  |  |  |  |  | Others |  |
| Finland (1998-1999) | AOM | 7m - 6y | 79 | 80% | *S. pneumoniae* | [[40](file:///C:\Users\s2820022\Desktop\The%20otopathogens%20review%201.xlsx#RANGE!_ENREF_121)] |
|  |  |  |  |  | *H. influenzae* |  |
|  |  |  |  |  | *M. catarrhalis* |  |
|  |  |  |  |  | *S. pyogenes* |  |
|  |  |  |  |  | *S. aureus* |  |
|  |  |  |  |  | *P. aeruginosa* |  |
|  |  |  |  |  | Others |  |
| Germany (2008-2010) | AOM | 3m - 5y | 24 | 42% | *H. influenzae* | [41] |
|  |  |  |  |  | *M. catarrhalis* |  |
|  |  |  |  |  | *S. aureus* |  |
|  |  |  |  |  | *A. Iwoffii* |  |
| ***Asia*** |  |  |  |  |  |  |
| Isreal (1995-1999) | AOM | ≤ 2m | 137 | 80% | *S. pneumoniae* | [[43](file:///C:\Users\s2820022\Desktop\The%20otopathogens%20review%201.xlsx#RANGE!_ENREF_140)] |
|  |  |  |  |  | *H. influenzae* |  |
|  |  |  |  |  | *M. catarrhalis* |  |
|  |  |  |  |  | *S. pyogenes* |  |
|  |  |  |  |  | Others |  |
| Isreal (1995-1996) | AOM | 3m - 3y | 249 | 73% | *H. influenzae* | [42] |
|  |  |  |  |  | *S. pneumoniae* |  |
|  |  |  |  |  | *M. catarrhalis* |  |
|  |  |  |  |  | *S. pyogenes* |  |
| Israel (1996-2003) | AOM | 3m - 3y | 771 | 77% | *H. influenzae* | [44] |
|  |  |  |  |  | *S. pneumoniae* |  |
|  |  |  |  |  | *M. catarrhalis* |  |
|  |  |  |  |  | Others |  |
| Turkey (1998-2000) | AOM | 6m - 10y | 78 | 56% | *S. pneumoniae* | [[51](file:///C:\Users\s2820022\Desktop\The%20otopathogens%20review%201.xlsx#RANGE!_ENREF_100)] |
|  |  |  |  |  | *H. influenzae* |  |
|  |  |  |  |  | *M. catarrhalis* |  |
|  |  |  |  |  | *S. pyogenes* |  |
|  |  |  |  |  | *S. aureus* |  |
|  |  |  |  |  | Others |  |
|  |  |  |  |  | *H. influenzae* |  |
|  |  |  |  |  | *M. catarrhalis* |  |
| Turkey (2002-2004) | AOM | 6m - 12y | 180 | 60% | *S. pneumoniae* | [52] |
|  |  |  |  |  | *H. influenzae* |  |
|  |  |  |  |  | *M. catarrhalis* |  |
|  |  |  |  |  | *S. pyogenes* |  |
|  |  |  |  |  | *S. aureus* |  |
|  |  |  |  |  | Others |  |
| Turkey (2003-2004) | AOM | 6m - 12y | 120 | 54% | *S. pneumoniae* | [53] |
|  |  |  |  |  | *H. influenzae* |  |
|  |  |  |  |  | *M. catarrhalis* |  |
|  |  |  |  |  | *S. pyogenes* |  |
|  |  |  |  |  | *S. aureus* |  |
|  |  |  |  |  | Others |  |
| Japan (1979-1980) | AOM | ≤ 16y | 406 | N/A | *S. pneumoniae* | [45] |
|  |  |  |  |  | *H. influenzae* |  |
|  |  |  |  |  | *S. pyogenes* |  |
|  |  |  |  |  | *S. aureus* |  |
|  |  |  |  |  | *P. aeruginosa* |  |
|  |  |  |  |  | Others |  |
| Japan (2002-2004) | AOM | < 10y | 1092 | 43% | *S. pneumoniae* | [48] |
|  |  |  |  |  | *H. influenzae* |  |
|  |  |  |  |  | *M. catarrhalis* |  |
| Japan (2003) | AOM | Children | 138 | 51% | *H. influenzae* | [47] |
|  |  |  |  |  | *S. pneumoniae* |  |
|  |  |  |  |  | *M. catarrhalis* |  |
| Taiwan (2004) | AOM | 4m - 13y | 96 | 49% | *S. pneumoniae* | [49] |
|  |  |  |  |  | *H. influenzae* |  |
|  |  |  |  |  | *S. aureus* |  |
|  |  |  |  |  | Others |  |
| Japan (2006) | AOM | 9m - 8y | 40 | 25% | *S. pneumoniae* | [18] |
|  |  |  |  |  | *H. influenzae* |  |
|  |  |  |  |  | *M. catarrhalis* |  |
|  |  |  |  |  | *S. pyogenes* |  |
|  |  |  |  |  | *S. aureus* |  |
| Thailand (2008-2009) | AOM | 3m - 5y | 107 | 48% | *S. pneumoniae* | [50] |
|  |  |  |  |  | *H. influenzae* |  |
|  |  |  |  |  | *M. catarrhalis* |  |
|  |  |  |  |  | *S. pyogenes* |  |
| ***Africa*** |  |  |  |  |  |  |
| South Africa (1999) | AOM | 2m - 7y | 173 | 27% | *S. pneumoniae* | [[54](file:///C:\Users\s2820022\Desktop\The%20otopathogens%20review%201.xlsx#RANGE!_ENREF_67)] |
|  |  |  |  |  | *H. influenzae* |  |
|  |  |  |  |  | *M. catarrhalis* |  |
|  |  |  |  |  | *S. pyogenes* |  |
|  |  |  |  |  | *S. aureus* |  |
|  |  |  |  |  | *P. aeruginosa* |  |
| **Average** |  |  |  | **62%** |  |  |
| **Max** |  |  |  | 95% |  |  |
| **Min** |  |  |  | 25% |  |  |
