# Supplementary material for: Predominant Bacteria Detected from the Middle Ear Fluid of Children Experiencing Otitis Media: A Systematic Review
Source: PLoS One. 2016 Mar 8;11(3):e0150949. doi: 10.1371/journal.pone.0150949 (PMC4783106; doi:10.1371/journal.pone.0150949)
Supplement: S2 Table — (DOCX) [file pone.0150949.s008.docx]

**S2 Table. Proportion of bacteria detected from MEF samples of patients with RAOM/AOMTF.**

| Countries | **OM** | **Age** | **Size** | **Positive for bacteria** | **Bacteria** | **Ref.** |
| --- | --- | --- | --- | --- | --- | --- |
| Costa Rica (1999-2001) | RAOM | 4m - 11y | 98 | N/A | *S. pneumoniae* | [16] |
|  |  |  |  |  | *H. influenzae* |  |
|  |  |  |  |  | *M. catarrhalis* |  |
|  |  |  |  |  | *S. pyogenes* |  |
| Costa Rica (1999-2001) | AOMTF | 4m - 11y | 76 | N/A | *H. influenzae* | [16] |
|  |  |  |  |  | *S. pneumoniae* |  |
|  |  |  |  |  | *M. catarrhalis* |  |
|  |  |  |  |  | *S. pyogenes* |  |
| Costa Rica (2002-2007) | RAOM | 2m - 8y | 138 | 57% | *S. pneumoniae* | [17] |
|  |  |  |  |  | *H. influenzae* |  |
|  |  |  |  |  | *M. catarrhalis* |  |
|  |  |  |  |  | *S. pyogenes* |  |
| Costa Rica (2002-2007) | AOMTF | 2m - 8y | 90 | 52% | *H. influenzae* | [17] |
|  |  |  |  |  | *S. pneumoniae* |  |
|  |  |  |  |  | *M. catarrhalis* |  |
|  |  |  |  |  | *S. pyogenes* |  |
| France (2007-2009) | RAOM/ AOMTF | 3m - 3y | 143* | 65% | *H. influenzae* | [55] |
|  |  |  |  |  | *S. pneumoniae* |  |
|  |  |  |  |  | *M. catarrhalis* |  |
|  |  |  |  |  | *T. otitidis* |  |
|  |  |  |  |  | *S. aureus* |  |
| Netherlands (2008-2009) | RAOM | < 6y | 25 | 28% | *H. influenzae* | [19] |
|  |  |  |  |  | *S. pneumoniae* |  |
|  |  |  |  |  | *M. catarrhalis* |  |
| Spain (2008-2010) | RAOM/ AOMTF | 3m - 3y | 77 | 68% | *H. influenzae* | [13] |
|  |  |  |  |  | *S. pneumoniae* |  |
|  |  |  |  |  | *M. catarrhalis* |  |
|  |  |  |  |  | *S. pyogenes* |  |
|  |  |  |  |  | *S. aureus* |  |
|  |  |  |  |  | Others |  |
| Australia (2007-2009) | RAOM | 7m - 3y | 143 | 27% | *H. influenzae* | [56] |
|  |  |  |  |  | *S. pneumoniae* |  |
|  |  |  |  |  | *M. catarrhalis* |  |
|  |  |  |  |  | *S. aureus* |  |
|  |  |  |  |  | *P. aeruginosa* |  |
|  |  |  |  |  | Others |  |
| Australia (2007-2009) | RAOM | 9m - 3y | 38 | 29% | *H. influenzae* | [57] |
|  |  |  |  |  | *S. pneumoniae* |  |
|  |  |  |  |  | *S. aureus* |  |
|  |  |  |  |  | *A. otitidis* |  |
| New Zealand (2011) | RAOM/OME (> 60% RAOM) | 6m-36m | 325 | 31% | H. influenzae | [58] |
|  |  |  |  |  | *S. pneumoniae* |  |
|  |  |  |  |  | *M. catarrhalis* |  |
|  |  |  |  |  | *A. otitidis* |  |
|  |  |  |  |  | *Others* |  |
| **Average** |  |  |  | **45%** |  |  |
| **Max** |  |  |  | 68% |  |  |
| **Min** |  |  |  | 27% |  |  |
