# Supplementary material for: Predominant Bacteria Detected from the Middle Ear Fluid of Children Experiencing Otitis Media: A Systematic Review
Source: PLoS One. 2016 Mar 8;11(3):e0150949. doi: 10.1371/journal.pone.0150949 (PMC4783106; doi:10.1371/journal.pone.0150949)
Supplement: S3 Table — (DOCX) [file pone.0150949.s009.docx]

**S3 Table. Proportion of bacteria detected from MEF samples of patients with OME/COME**

| **Countries/regions** | **OM** | **Age** | **Size** | **Positive for bacteria** | **Bacteria** | **Ref.** |
| --- | --- | --- | --- | --- | --- | --- |
| ***America*** |  |  |  |  |  |  |
| Brazil (2001-2002) | OME/ COME | 11m - 10y | 128 | 25% | *H. influenzae* | [59] |
|  |  |  |  |  | *S. pneumoniae* |  |
|  |  |  |  |  | *M. catarrhalis* |  |
|  |  |  |  |  | Others |  |
| The US (1995) | COME | 9m -15y | 97 | 29% | *H. influenzae* | [60] |
|  |  |  |  |  | *S. pneumoniae* |  |
|  |  |  |  |  | *M. catarrhalis* |  |
| ***Europe*** |  |  |  |  |  |  |
| Finland (1981) | COME | 5m - 15y | 110 | 32% | *H. influenzae* | [61] |
|  |  |  |  |  | *S. pneumoniae* |  |
|  |  |  |  |  | *M. catarrhalis* |  |
|  |  |  |  |  | *S. aureus* |  |
|  |  |  |  |  | Others |  |
| Wales (1986-1987) | COME | ≤ 7y (67%) | 259 | 20% | *H. influenzae* | [62] |
|  |  |  |  |  | *S. pneumoniae* |  |
|  |  |  |  |  | *M. catarrhalis* |  |
|  |  |  |  |  | *S. aureus* |  |
| England (1989) | COME | < 10y (Mostly) | 102 | 47% | *H. influenzae* | [63] |
|  |  |  |  |  | *S. pneumoniae* |  |
|  |  |  |  |  | *M. catarrhalis* |  |
|  |  |  |  |  | Others |  |
| Finland (1993-1994) | OME | 5m - 12y | 165 | 39% | *S. pneumoniae* | [64] |
|  |  |  |  |  | *H. influenzae* |  |
|  |  |  |  |  | *M. catarrhalis* |  |
|  |  |  |  |  | *S. pyogenes* |  |
|  |  |  |  |  | *S. aureus* |  |
|  |  |  |  |  | others |  |
| Finland (1993-1994) | COME | < 12y | 123 | 45% | *H. influenzae* | [65] |
|  |  |  |  |  | *S. pneumoniae* |  |
|  |  |  |  |  | *M. catarrhalis* |  |
| Finland (1996-1997) | OME | 1y - 9y | 67 | 21% | *H. influenzae* | [66] |
|  |  |  |  |  | *M. catarrhalis* |  |
|  |  |  |  |  | *S. pneumoniae* |  |
| Spain (2007) | COME | 1y - 12y | 40 | 73% | *H. influenzae* | [67] |
|  |  |  |  |  | *S. pneumoniae* |  |
|  |  |  |  |  | *S. aureus* |  |
|  |  |  |  |  | *A. otitidis* |  |
| Netherlands (2008-2009) | COME | < 6y | 94 | 32% | *H. influenzae* | [19] |
|  |  |  |  |  | *M. catarrhalis* |  |
|  |  |  |  |  | *S. pneumoniae* |  |
| UK (2012) | COME | ≤ 18y (83%) | 62 | 45% | *S. pneumoniae* | [68] |
|  |  |  |  |  | *M. catarrhalis* |  |
|  |  |  |  |  | *H. influenzae* |  |
|  |  |  |  |  | *S. aureus* |  |
|  |  |  |  |  | others |  |
| ***Asia*** |  |  |  |  |  |  |
| Turkey (1999) | COME | 2y - 14y | 37 | 24% | *S. pneumoniae* | [69] |
|  |  |  |  |  | *H. influenzae* |  |
|  |  |  |  |  | *M. catarrhalis* |  |
| Korea (2004-2008) | COME | 2y - 7y | 289 | 36% | *S. pneumoniae* | [70] |
|  |  |  |  |  | *S. pyrogenes* |  |
|  |  |  |  |  | *S. aureus* |  |
|  |  |  |  |  | *P. aerigunosa* |  |
|  |  |  |  |  | Others |  |
| Korea (2000-2002) | COME | 1y - 11y | 278 | 14% | *H. influenzae* | [71] |
|  |  |  |  |  | *S. pneumoniae* |  |
|  |  |  |  |  | *S. pyrogenes* |  |
|  |  |  |  |  | *S. aureus* |  |
| Japan (1988) | OME | ≤ 15y (73%) | 613 | 42% | *H. influenzae* | [72] |
|  |  |  |  |  | *S. pneumoniae* |  |
|  |  |  |  |  | *M. catarrhalis* |  |
|  |  |  |  |  | *S. aureus* |  |
|  |  |  |  |  | Others |  |
| Japan (2006) | COME | 9m - 8y | 76 | 21% | *H. influenzae* | [18] |
|  |  |  |  |  | *S. pneumoniae* |  |
|  |  |  |  |  | *M. catarrhalis* |  |
|  |  |  |  |  | *S. aureus* |  |
| Iran (2007-2008) | COME | 2y - 13y | 63 | 60% | *S. pneumoniae* | [73] |
|  |  |  |  |  | *H. influenzae* |  |
|  |  |  |  |  | *M. catarrhalis* |  |
|  |  |  |  |  | *S. aureus* |  |
|  |  |  |  |  | *P. aeruginosa* |  |
|  |  |  |  |  | Others |  |
| Iran (2009-2010) | COME | 1y - 12y | 63 | 48% | *S. pneumoniae* | [74] |
|  |  |  |  |  | *M. catarrhalis* |  |
|  |  |  |  |  | *H. influenzae* |  |
|  |  |  |  |  | *A. otitidis* |  |
| Lebanon (1996-1997) | OME | 2y - 10y | 47 | 21% | *H. influenzae* | [75] |
|  |  |  |  |  | *M. catarrhalis* |  |
| Lebanon (2009-2010) | COME | < 13y | 107 | 32% | *H. influenzae* | [76] |
|  |  |  |  |  | *S. pneumoniae* |  |
|  |  |  |  |  | *M. catarrhalis* |  |
| ***Africa*** |  |  |  |  |  |  |
| Egypt (1993) | COME | 15m - 8y | 104 | 50% | *H. influenzae* | [77] |
|  |  |  |  |  | *M. catarrhalis* |  |
|  |  |  |  |  | *S. pneumoniae* |  |
|  |  |  |  |  | *S. aureus* |  |
|  |  |  |  |  | *Pseudomonas* spp. |  |
| Egypt (2003-2008) | OME/COME | 3y - 10y | 50 | 24% | *S. pneumoniae* | [78] |
|  |  |  |  |  | *M. catarrhalis* |  |
| ***Oceania*** |  |  |  |  |  |  |
| Australia (1995-2000) | COME | 11m - 10y | 45 | 42% | *H. influenzae* | [79] |
|  |  |  |  |  | *M. catarrhalis* |  |
|  |  |  |  |  | *S. aureus* |  |
|  |  |  |  |  | *P. aeruginosa* |  |
|  |  |  |  |  | Others |  |
| New Zealand (1994) | COME | 11m - 8y | 105 | 36% | *H. influenzae* | [80] |
|  |  |  |  |  | *M. catarrhalis* |  |
|  |  |  |  |  | *S. pneumoniae* |  |
|  |  |  |  |  | Others |  |
| **Average** |  |  |  | **36%** |  |  |
| **Max** |  |  |  | 73% |  |  |
| **Min** |  |  |  | 14% |  |  |
